# Effect of High‐Power LED Barriers on Shear Bond Strength and Curing Time in Orthodontic Brackets With Single‐Component Adhesive

**DOI:** 10.1002/cre2.70153

**Published:** 2025-06-11

**Authors:** Yasaman Bozorgnia, Mahla Tak, Maryam Jamali

**Affiliations:** ^1^ Department of Orthodontics, School of Dentistry North Khorasan University of Medical Sciences Bojnurd Iran; ^2^ Student, School of Dentistry North Khorasan University of Medical Sciences Bojnurd Iran; ^3^ Department of Restorative and Aesthetic Dentistry, School of Dentistry North Khorasan University of Medical Sciences Bojnurd Iran

**Keywords:** infection control, light emitting diodes, shear bond strength, single component adhesive

## Abstract

**Objective:**

The dental light‐curing unit is a crucial component in bonding brackets. Since it cannot be steam‐sterilized, it is covered with a disposable barrier and disinfected between patients. This study examined the impact of the cellophane layer used to cover the light guides on both the shear bond strength and the adhesive remnant index.

**Material and Methods:**

Fifty human premolars, extracted for clinical purposes, were randomly allocated into five groups. Three of these groups were cured using a high‐power LED unit with a cellophane layer, applying curing times of 3, 6, and 9 s. The remaining two groups were cured for 3 and 6 s without a cellophane layer. The bracket shear bond strengths were assessed utilizing a universal testing machine. The collected data underwent analysis through a one‐way analysis of variance (ANOVA), followed by Tukey's post hoc test.

**Results:**

The group with the highest shear bond strength was group 5 (6 s of light curing without a cellophane layer), while group 1 (3 s of light curing with a cellophane layer) had the lowest shear bond strength. Significant statistical differences were identified among the groups (*p* < 0.05). Moreover, no notable differences were observed concerning the adhesive remnant index.

**Conclusion:**

The cellophane layer decreased the shear bond strength. Therefore, to achieve clinically accepted values, at least 9 s of radiation is necessary when using cellophane.

## 1 Introduction

Esthetics play a vital role in contemporary dental practice, especially in orthodontics, where increasing numbers of adult patients are seeking treatment to improve their appearance (Lyons et al. 2019). According to a 2015 report by the American Association of Orthodontists, the number of adult orthodontic patients nearly doubled between 2010 and 2014 (Alzainal et al. 2020). While esthetics remain a primary motivator, the long‐term success of orthodontic therapy also depends on the durability and safety of the adhesive interface between the brackets and the enamel surface (Cerekja and Cakirer 2011; Mohammed et al. 2023; Rohini et al. 2023; Shapinko et al. 2018). Effective bracket bonding is essential for maintaining treatment progress and avoiding clinical complications such as bond failure, enamel damage during debonding, and increased chair time (Bakhadher et al. 2015). The adhesive must provide adequate shear bond strength (SBS) to withstand masticatory and orthodontic forces throughout treatment (Alzainal et al. 2020; Gomes et al. 2014). Reynolds (1975) suggested that a clinically acceptable SBS ranges from 5.9 to 7.8 MPa. Additionally, the adhesive should facilitate clean debonding with minimal enamel damage, preserving tooth integrity (Asefi et al. 2021).

Light‐curing units (LCUs) and resin‐based adhesives are central to the success of bonding procedures. The degree of conversion of light‐activated composites is influenced by light intensity, cure time, and material composition. Since light intensity and cure time are inversely related, it is theoretically possible to compensate for a decrease in one by increasing the other (Gomes et al. 2014; Halvorson et al. 2002). Modern high‐power LED LCUs have enabled reduced exposure times while maintaining sufficient curing depth and bond strength (Agrawal 2013; Mirabella et al. 2008). Technological advancements have led to the development of third‐generation LEDs with output intensities of over 1000 mW/cm², allowing exposure times as short as 3–5 s (Almeida et al. 2018; Gomes et al. 2014; Pelissier et al. 2011; Rohini et al. 2023).

In parallel, newer adhesive systems such as GC Ortho Connect have been introduced, offering enhanced physical properties, fluoride release, and simplified application protocols (Joseph et al. 2022; Perković et al. 2023; Shalini et al. 2023; Shapinko et al. 2018). GC Ortho Connect offers high translucence, excellent esthetics, stain resistance, and fluoride content that helps reduce enamel demineralization. Its handling ease and built‐in fluorescence also assist with cement cleanup during bracket placement and removal (Asefi et al. 2021; Esmaily et al. 2023; Joseph et al. 2022; Ok et al. 2021). This self‐adhesive material reduces technique sensitivity and eliminates the need for separate bonding agents, streamlining clinical workflow (Al‐Salem et al. 2024)

From an infection control standpoint, the light guide of the curing unit is classified as a semi‐critical instrument due to its contact with mucosal tissues. Proper disinfection or barrier protection is therefore essential to prevent cross‐contamination (Khode et al. 2017; Soares et al. 2020; Caughman et al. 1989; Nahidh et al. 2022; Nelson et al. 1999; Rueggeberg et al. 1996; Scott et al. 2004; Soares et al. 2020).

Although previous research has evaluated the effect of barrier sleeves and different adhesive systems independently, limited data exist on the combined influence of high‐power LED curing units, barrier films, and new self‐adhesive composites on orthodontic bond strength. Therefore, this study aimed to investigate the impact of a translucent cellophane barrier on the shear bond strength and adhesive remnant index (ARI) when bonding orthodontic brackets using a high‐power LED unit and GC Ortho Connect adhesive.

## 2 Materials and Methods

This in vitro study was conducted on 50 human premolars extracted for orthodontic purposes. The included teeth had intact buccal enamel surfaces with no caries, cracks, restorations, or developmental defects. Teeth with enamel developmental defects not visible macroscopically but suspected under magnification, or those with surface irregularities after polishing, were excluded. The study protocol was approved by the Ethics Committee of North Khorasan University of Medical Sciences (IR.NKUMS.REC.1403.054), and verbal informed consent was obtained from all donors or their legal guardians.

The sample size was determined using the following formula based on the previous study by Nahidh et al. (2022):

$$n=2{\left(\sigma \frac{Z_{1-\frac{\alpha }{2\tau }}+Z_{1-\beta }}{\mu_{A}-\mu_{B}}\right)}^{2}$$

where *n* is the required sample size, α = 0.05, power = 95%, *σ* is the standard deviation, *τ* is the number of repetitions, and *μA − μB* is the minimum expected difference in means. Based on this formula, the minimum calculated sample size was 6 teeth per group; however, to enhance the reliability and robustness of the results, 10 teeth were allocated to each group. Samples were stored in sterile saline at 4°C until testing.

**Figure 1:**
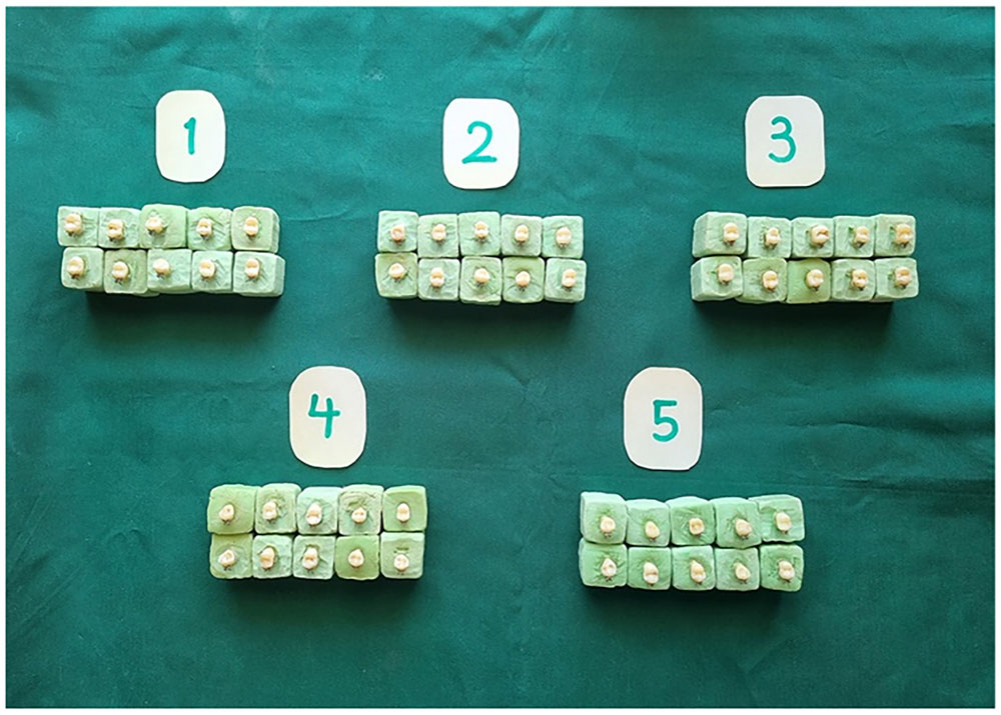
Mounted specimens with instant self‐cure acrylic RE (Acropars, Iran). The crown of the tooth is immersed in acrylic up to the CEJ.

Soft tissue remnants were removed, and the buccal surfaces were polished using non‐fluoridated pumice (Sina, Iran) and rubber prophylactic cups at low speed for 10 s. The samples were then stored in isotonic normal saline (0.90% w/v NaCl, 300 mosM/L) to avoid any impact of residual solutions on bonding efficacy (Heravi et al. 2013). Each tooth was mounted in cold‐cure acrylic resin (Acropars 200, Iran) in 2 × 2 × 2 cm blocks, with the root extending 1 mm beyond the cementoenamel junction (CEJ), and the buccal surface was aligned parallel to the debonding force (Figure 1). The enamel surfaces were etched using 37% phosphoric acid gel (Morvabon, Iran) for 30 s, rinsed with air–water spray for 20 s, and air‐dried using oil‐ and moisture‐free air until a frosty white appearance was obtained.

Orthodontic brackets (Opal/Cast MBT 0.022 brackets by Hilal T.S.H, Iran; base area = 11.34 mm²) were bonded using GC Ortho Connect light‐cure adhesive resin (GC Corporation, Japan). The adhesive was applied to the base of each bracket, positioned on the tooth surface, and excess resin was removed using a scaler. All bonding procedures were carried out by a single orthodontist to ensure consistency and reduce operator‐related variability.

A high‐power LED curing unit (Woodpecker LED.H Ortho Cure, China) was used to cure the adhesive, maintaining a vertical position at a 2 mm distance from the bracket. The unit delivered an irradiance of 1200 mW/cm² in high‐power mode. In all experimental groups, a thin cellophane layer was placed over the curing tip as a protective barrier to prevent cross‐contamination, as explained in the Introduction. The samples were randomly divided into five groups (*n* = 10), based on light‐curing protocol and whether or not a protective cellophane barrier.

- Group 1 (experimental): 3 s of curing from the occlusal surface using the high‐power mode with Cellophane Layer.
- Group 2 (experimental): 6 s total (3 s from occlusal + 3 s from mesial surface) using the high‐power mode with Cellophane Layer.
- Group 3 (experimental): 9 s total (3 s from each occlusal, mesial, and distal surface) using high‐power mode with Cellophane Layer.
- Group 4 (control): 3 s of curing from the occlusal surface using high‐power mode without Cellophane Layer.
- Group 5 (control): 6 s total (3 s from occlusal + 3 s from mesial surface) using high‐power mode without Cellophane Layer.

### 2.1 Testing of Shear Bond Strength

**Figure 2:**
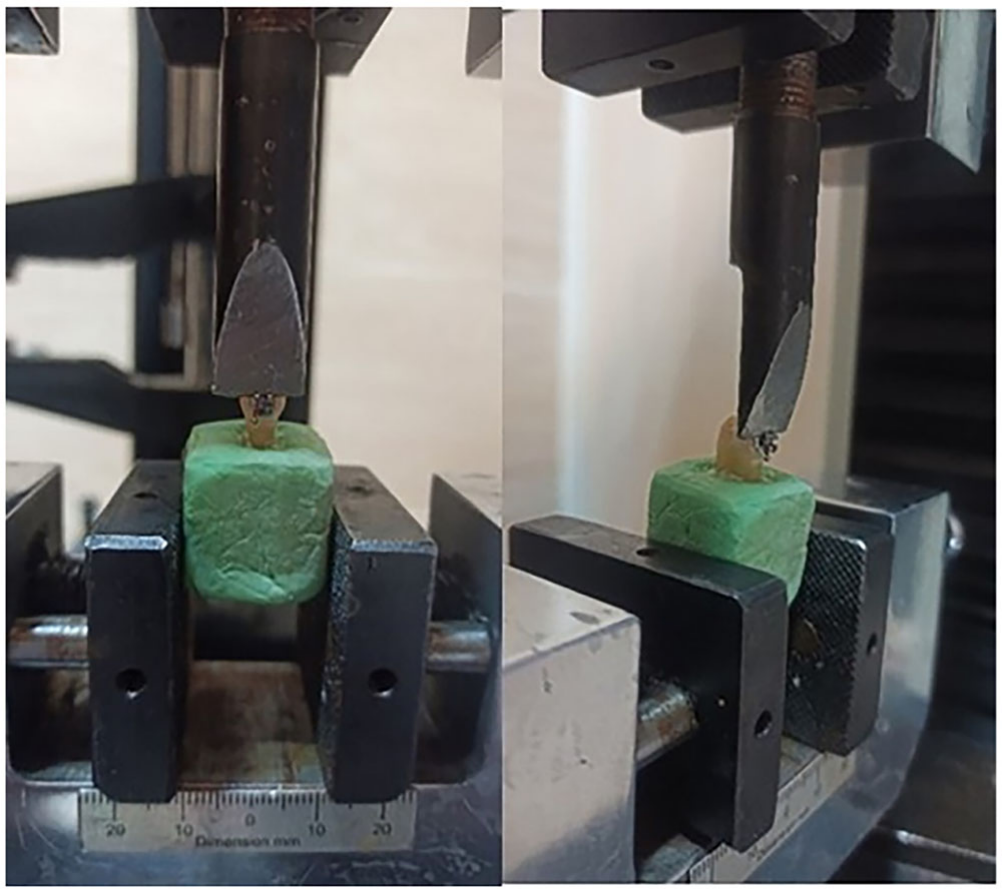
An image of a sample fixed on the universal testing machine device.

After bonding, samples were stored in separate containers with 0.9% normal saline at 37°C in a dark environment for 24 h. The SBS was measured using a universal testing machine (STM20, Santam, Iran) with a crosshead speed of 1 mm/min (Figure 2). The SBS values were recorded in megapascals (MPa).

### 2.2 Testing the ARI

Following bracket debonding, enamel surfaces were examined under a stereomicroscope at 10× magnification to assess the ARI, based on the classification by Bishara et al. (2000):

Score 5: No adhesive remains on enamel.

Score 4: < 10% of adhesive remains.

Score 3: 10%–90% of adhesive remains.

Score 2: > 90% of adhesive remains.

Score 1: All adhesive remains, including bracket base impression.

This evaluation was used to determine the predominant mode of bond failure.

### 2.3 Statistical Analysis

Data were analyzed using IBM SPSS Statistics for Windows (version 23.0, Armonk, NY: IBM Corp, 2015). The Shapiro–Wilk test was performed to assess data normality. A one‐way analysis of variance (ANOVA), followed by Tukey's post hoc test, was used to compare SBS values across groups. The ARI scores, as categorical variables, were analyzed using the Kruskal–Wallis test. No statistically significant differences were observed between the groups.

### 2.4 Use of Large Language Models (LLMs)

As English is not our first language, we relied on ChatGPT, an AI language model, to assist with grammar, punctuation, spelling, and overall language refinement to improve clarity and linguistic accuracy.

## 3 Results

The data's normality was first evaluated using the Shapiro‐Wilk test, which confirmed a normal distribution (*p* > 0.05). This result validated the application of parametric tests. Subsequently, a one‐way ANOVA test identified a significant variation in shear bond strength across the groups (F (4, 45) = 6.827, *p* < 0.05). As shown in Table 1, Tukey's post hoc tests indicated no significant differences between Groups 1 and 2, or 4 and 5. However, significant differences were observed when Groups 1 and 2 were compared to Groups 4 and 5. Group 3 showed no significant difference when compared to the other groups. Therefore, Group 3 can be considered a suitable alternative to Groups 4 and 5.

**Table 1:** Tukey's post hoc test.

Group	*N*	Subset for alpha = 0.05	
		1	2
Group 1	10	5.4140	
Group 2	10	5.5120	
Group 3	10	7.0269	7.0269
Group 4	10		10.6340
Group 5	10		10.9290
Sig.		0.799	0.071

*Note:* Means for groups in homogeneous subsets are displayed.

Uses Harmonic Mean Sample Size = 10.000. Group 1: 3 s light curing with CL (Cellophane Layer); Group 2: 6 s light curing with CL; Group 3: 9 s light curing with CL; Group 4 (control) 3 s light curing without CL; Group 5 (control): 6 s light curing without CL.

**Figure 3:**
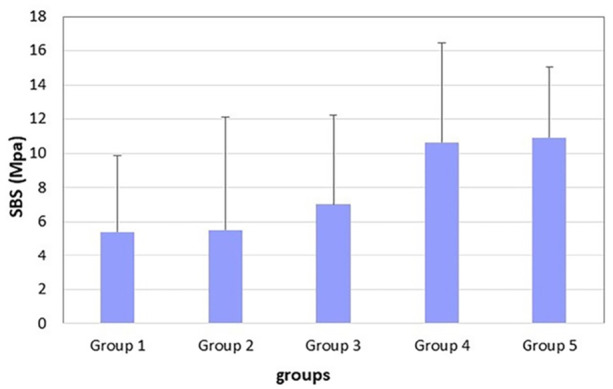
Mean values of shear bond strength (MPa) according to several experimental groups.

The mean shear bond strength values are presented in Table 2. The highest mean bond strength was observed in Group 5, which had a 6‐second curing time without the cellophane layer. In contrast, the lowest mean bond strength was found in Group 1, where a 3‐second curing time was used with the cellophane layer. According to Reynolds' (1975) study, there was no significant difference in SBS values between the two control groups, both of which showed SBS values above the clinically accepted levels. These control groups also exhibited values that were significantly higher than those in the experimental groups. Only Group 3 demonstrated sufficient SBS values for orthodontic bonding among the three experimental groups, as illustrated in Figure 3.

**Table 2:** Descriptive statistics.

Groups	N	Minimum	Maximum	Mean	Std. Deviation
Group 1	10	0.94	9.89	5.4140	3.14386
Group 2	10	2.09	12.64	5.5620	2.89176
Group 3	10	2.52	12.25	7.0269	3.20959
Group 4	10	4.71	16.48	10.6340	2.89194
Group 5	10	4.00	15.04	10.9290	4.03591

*Note:* Group 1: 3 s light curing with CL (Cellophane Layer); Group 2: 6 s light curing with CL; Group 3: 9 s light curing with CL; Group 4 (control) 3 s light curing without CL; Group 5 (control): 6 s light curing without CL. The highest mean bond strength was seen in Group 5 and the lowest value was in Group 1.

**Figure 4:**
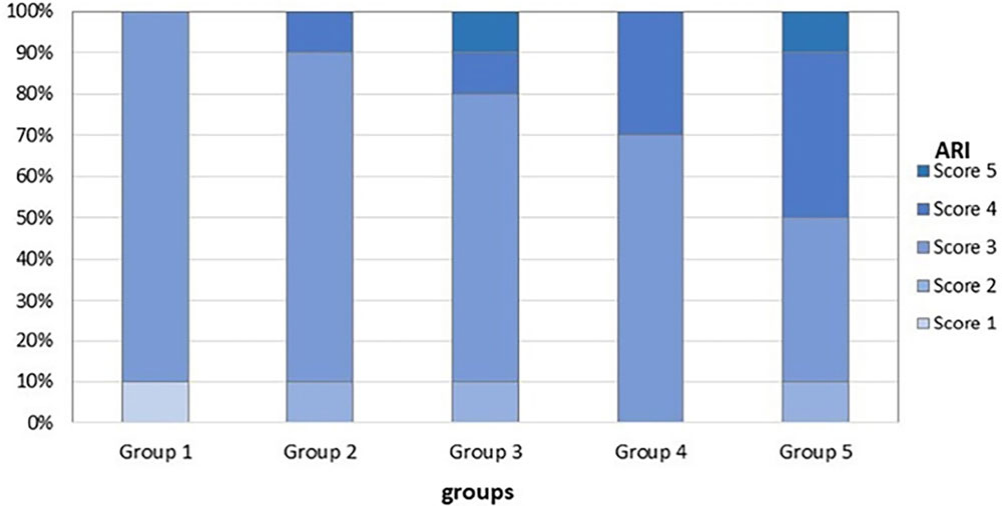
Adhesive Remnant Index (ARI) scores distribution by experimental groups.

The ARI scores are shown in Table 3. The Chi‐square test indicated no statistically significant differences among the groups (Figure 4).

**Table 3:** Group * ARI crosstabulation count.

Groups	ARI					Total
	1	2	3	4	5	
Group 1	1	0	9	0	0	10
Group 2	0	1	8	1	0	10
Group 3	0	1	7	1	1	10
Group 4	0	0	7	3	0	10
Group 5	0	1	4	4	1	10
Total	1	3	35	9	2	50

*Note:* Group 1: 3 s light curing with CL (Cellophane Layer); Group 2: 6 s light curing with CL; Group 3: 9 s light curing with CL; Group 4 (control) 3 s light curing without CL; Group 5 (control): 6 s light curing without CL. The ARI score of 3 has the highest frequency in all the experimental groups, the score of 1 has the lowest frequency.

## 4 Discussion

The shear bond strength of orthodontic brackets depends on some factors such as tooth preparation, adhesive system, bracket base design, and the method of curing (Guram and Shaik 2018). Numerous authors say it is possible to calculate the amount of light required to sufficiently cure a resin composite through the multiplication of the light intensity and exposure time. According to this principle, a single second of exposure is enough to use a curing device with an output of 1600 mW/cm². However, in a study by Gomes et al. (2014) a 2‐second exposure was found to be inadequate for achieving optimal bond strength. Conversely, a study by Rohini et al. (2023) have found that the shear bond strength for groups with the curing time of 1, 3, and 6 s using the Woodpecker I LED with the output 2400 mW/cm² was 10.69, 11.58, and 16.04 MPa, respectively, which went beyond the ideal measures of Reynolds (1975) of 5.9–7.8 MPa. In the study by Almeida et al. (2018) shear bond strength was found to be 15.79 and 21.57 MPa for output times of 3 and 6 s when an LED light with an irradiance of 3200 mW/cm² was used. In favor of our study, Al‐Salem et al. (2024) showed that the shear bond strength of the self‐adhesive (GC Ortho Connect) with a curing time of 3 s acquired an average value of 10.20 MPa.

In clinical practice, increased shear bond strength can pose additional challenges during bracket removal, particularly in cases that require early debonding. This situation may raise the risk of enamel damage and cause discomfort for the patient. Therefore, it is essential to choose an appropriate curing time that balances the benefits of improved bond strength with the potential clinical risks. As a result, the shear bond strength achieved with a 3‐s cure time is considered more reliable and aligns more closely with findings from studies that use conventional light‐curing techniques.

While high‐power light‐curing devices considerably reduce curing time, they also involve higher initial costs and may require more maintenance. A cost–benefit analysis should be performed to assess whether the time savings from reduced chair time justify the added expenses, especially in clinics with high patient turnover, where such savings can lead to economic advantages.

In this study, we found that when the Cellophane layer was not used, light curing at an intensity of 1200 mW/cm² for durations of 3 and 6 s resulted in bond strengths of 10.64 and 10.93 MPa, respectively. Both values exceed the clinically acceptable threshold for bond strength, which is a crucial factor in adhesive selection.

Most studies examining the impact of LCU intensity on the shear bond strength of orthodontic brackets typically utilize Transbond XT adhesive, recognized as the gold standard for bonding (Rohini et al. 2023; Shapinko et al. 2018). Research on the effects of infection control barriers on LCUs also commonly employs Transbond XT (Nahidh et al. 2022). In contrast, our study uses GC Ortho Connect composite for bonding brackets. We favor GC Ortho Connect not only for its impressive shear bond strength results but also for its practical benefits, such as ease of use and reduced preparation time. However, it is important to note that the cost of this composite may be higher than that of other materials, which could affect the overall treatment expenses.

Bahrami et al. (2023) compared the shear bond strengths of three adhesive systems: Transbond XT without primer, Transbond XT with primer, and GC Ortho Connect composite. Their findings revealed bond strengths of 11.18, 12.66, and 15.50 MPa, respectively, indicating that GC Ortho Connect outperformed Transbond XT. Similarly, Asefi et al. (2021) tested three adhesives—self‐cured, light‐cured, and GC Ortho Connect—and found that GC Ortho Connect produced shear bond strengths within the clinically acceptable range, making it a viable alternative to other adhesives.

The superior performance of GC Ortho Connect in various studies suggests that newer adhesive formulations may provide enhanced bonding capabilities. However, practical implications such as the adhesive's viscosity, ease of application, and compatibility with different curing lights must be considered. Additionally, further investigation into the long‐term durability of these bonds under various oral conditions—such as moisture control and temperature fluctuations—is necessary to ensure consistent clinical outcomes.

According to the infection control guidelines established by the CDC (Centers for Disease Control and Prevention) and ADA (American Dental Association), all patients should be treated as potentially infectious. This requires strict adherence to instrument sterilization and surface disinfection protocols (AlShaafi 2013).

Various methods for infection control of light‐cure units have been implemented, each with its benefits and drawbacks. For example, glutaraldehyde‐based disinfectants are effective and quick for sterilizing light guide tips; however, repeated use may gradually degrade the optical fibers, reducing the device's performance. This degradation can lead to diminished light output, affecting composite materials' curing quality. Consequently, this might require more frequent maintenance or replacement of the equipment in the long run. Additionally, studies have shown that autoclaving significantly reduces the effectiveness of the light guide (AlShaafi 2013; Nahidh et al. 2022; Scott et al. 2004).

The use of a disposable infection control barrier over the light guide is the most practical and convenient method; however, it results in reduced light intensity from the LCU (Khode et al. 2017). Scott et al. (2004) found that among three different barriers, plastic wrap had the least impact on reducing the radiant power of the LCU. Since infection control barriers, such as cellophane layers, are not sterile, the potential risks of contamination during their use should be carefully considered. Additionally, the packaging and storage of these barriers in a clinical environment may increase the risk of contamination, particularly if they are packaged in bulk. Therefore, it is important to implement appropriate strategies to maintain sterility and minimize the risk of contamination.

Our findings align with those of Nahidh et al. (2022), who reported a 50% reduction in shear bond strength when infection control barriers were used. These results imply that clinicians should be aware of the potential negative impact of these barriers on treatment quality. They may need to consider extending curing times when necessary to ensure the materials cure completely. Further investigation into this issue could help refine clinical protocols.

The results also indicated that the protective barrier had no significant effect on the site of bond failure, as assessed by the ARI. Using Bishara's modified index, all groups scored between 3 and 4, suggesting that bond failure occurred primarily between the adhesive and the bracket, leaving more adhesive on the tooth surface. This finding reduces the risk of enamel damage but may require additional time for orthodontists to clean the residual composite, potentially causing discomfort for patients.

In addition to extending curing times, utilizing high‐power light‐curing devices may enhance patient comfort by minimizing the duration of procedures. However, it is crucial to consider patient sensitivity to light and heat, as prolonged exposure to high‐intensity light could cause discomfort or even minor thermal damage. Incorporating patient feedback and adjusting the curing protocol accordingly can help optimize the treatment experience.

The use of GC Ortho Connect composite, which eliminates the need for a bonding agent or primer, not only simplifies the bonding process but may also reduce the overall treatment time and associated costs of bonding materials. However, the impact of this simplification on long‐term bond strength and bracket stability should be further investigated to ensure that the streamlined process does not compromise the quality of treatment. Additional clinical trials could confirm that this new approach continues to deliver favorable outcomes across different clinical settings.

Our study found that using infection control barriers, such as the Cellophane layer on light‐curing devices— a common and cost‐effective method in dental clinics—can reduce the shear bond strength of orthodontic brackets. We observed that by increasing the curing time by just 3 s, the shear bond strength achieved fell within the ideal range, showing no significant difference when compared to the control groups.

In clinical settings, reduced shear bond strength may result in premature bracket failures, which could lead to repairs and repeated treatments. This issue affects the efficiency and cost of treatment and impacts the patient experience. Therefore, it is crucial to find a balance between the necessity of infection control and maintaining adequate shear bond strength.

## 5 Conclusion

The study demonstrated that using a cellophane layer for infection control on light‐curing devices can reduce the shear bond strength of orthodontic brackets by up to 50%, which may bring it below clinically acceptable levels. To compensate for this reduction, it is essential to increase the curing time. The findings indicate that extending the curing time to 9 s helps maintain bond strength within an acceptable range, even though it is still lower than that of the group without cellophane.

Therefore, when using infection control barriers on light‐curing devices, it is crucial to increase the exposure time to ensure sufficient bond strength and reduce the risk of bracket debonding during treatment.

## 6 Limitations of This Study

The present study has several limitations that future exploration should address. The in vitro approach used in this study does not fully capture the complexities of the oral environment, such as the presence of saliva, variations in oral hygiene, temperature fluctuations, and changes in pH levels. Furthermore, the relatively small sample size may limit the generalizability of the findings. To gain a more comprehensive understanding, it is important to conduct additional in vitro experiments that examine a wider range of bracket materials, adhesive systems, and infection control barriers from various manufacturers, employing different application protocols. Additionally, in vivo studies will be essential to validate these findings and assess their practical implications in clinical settings.

## Author Contributions

The work presented here was carried out in collaboration among all authors. Yasaman Bozorgnia designed the methods and experiments and selected bonding materials and devices, bonded the brackets, and interpreted the results. Mahla Tak collected and prepared the samples, carried out the SBS test, and drafted the manuscript. Maryam Jamali reviewed and edited the paper. All authors read and approved the final manuscript.

## Conflicts of Interest

The authors declare no conflicts of interest.

## Supporting information

Supporting Material Raw Force Measurements (Newtons).

## Data Availability

Data is available on request from the authors.
